# Evaluating the Impact of a Molecular Diagnostic Algorithm on Tuberculosis and Nontuberculous Mycobacterial Infections in Newfoundland and Labrador, Canada

**DOI:** 10.3390/biomedicines13102416

**Published:** 2025-10-02

**Authors:** Robert Needle, Yang Yu, Hafid Soualhine, Catherine Yoshida, Lei Jiao, Rodney Russell

**Affiliations:** 1 Division of Biomedical Sciences, Memorial University of Newfoundland, St. John’s, NL A1B 3V6, Canada; 2 NL Heath Services, Public Health and Microbiology Laboratory, St. John’s, NL A1A 3Z9, Canada; 3 Department of Pathology & Laboratory Medicine, University of Ottawa, Ottawa, ON K1H 8M5, Canada; 4 National Microbiology Laboratory, Public Health Agency of Canada, Winnipeg, MB R3E 3R2, Canada; 5 Department of Medical Microbiology and Infectious Diseases, University of Manitoba, Winnipeg, MB R3E 0T4, Canada

**Keywords:** tuberculosis, MTBC, diagnostics, NAAT, molecular assay

## Abstract

**Background/Objectives**: The diagnosis of *Mycobacterium tuberculosis* complex (MTBC) and nontuberculous mycobacterial (NTM) infections is accomplished by three main diagnostics methods: smear microscopy, culture, and molecular testing. Diagnostic algorithms used by laboratories can significantly impact clinical and infection control management. Current Canadian Tuberculosis Standards recommend the use of nucleic acid amplification testing (NAAT) for smear-positive patients and smear-negative patients upon request. An alternative algorithm is to utilize NAAT in the Panel approach on all samples, pulmonary and extrapulmonary, to potentially reduce time to diagnosis and treatment. This alternative approach was implemented in November 2019 at the Newfoundland and Labrador Public Health and Microbiology Laboratory (NL PHML) using a laboratory-developed multiplex real-time PCR (LDT m-qPCR) assay targeting *Mycobacterium* spp. (*Myco* spp.) and MTBC, performed in parallel with smear and culture. **Methods:** To investigate the impact of this alternate testing approach, we conducted an observational retrospective analysis of laboratory diagnostic and treatment data, recognizing that temporal changes in epidemiology, clinical practice, and laboratory workflow may also have influenced outcomes. To complete this, study data from three years before and four years after implementation were gathered. **Results**: The sensitivity/specificity of the smear, m-LDT qPCR-MTBC, m-LDT qPCR-*Myco* spp., and culture assays in this study were 18.1%/100%, 96.7%/99.8%, 47.6%/99.0%, and 96.8%/100%, respectively. The gold standard utilized for these calculations was clinical diagnosis for active MTBC disease and culture for NTM infections, recognizing that the use of clinical diagnosis may introduce subjectivity. The Panel approach reduced the time to diagnosis of tuberculosis MTBC by 29 days (*p* < 0.0001) for NL PHML, and when modelled for a laboratory with rapid culture identification, diagnosis was reduced by 14 days (*p* = 0.003). Among non-empirically treated tuberculosis patients, the time to treatment was decreased by 25.5 days (*p* < 0.001). For NTM infections, rapid diagnostics only affected one patient’s treatment. This finding agrees with clinical management guidelines, which do not routinely utilize rapid diagnostics for the diagnosis of disease or treatment decisions. The cost implications of additional NAAT testing were calculated to be an increase of CAD 23.62 per sample. **Conclusions**: Our findings support the adoption of a molecular assay for MTBC as an initial diagnostic tool to decrease time to diagnosis and time to treatment, depending on local epidemiology and irrespective of smear status. Utilizing a molecular assay for genus level identification of NTM had minimal impact on clinical management suggesting its limited diagnostic utility in a broad population setting.

## 1. Introduction

*Mycobacterium tuberculosis,* a member of the *Mycobacterium tuberculosis* complex (MTBC), is the causative agent of tuberculosis (TB) infection, which presents many diagnostic, clinical, and public health challenges. In 2023, TB re-emerged as the world’s most common cause of death from a single infectious agent [1]. TB can cause severe lung damage, leading to respiratory failure and disseminated infection involving multiple organs, including bone, the central nervous system, genitourinary tract, and other organs. Without appropriate treatment, the mortality rate can be as high as 50% without treatment [2]. Globally, TB has an estimated incidence of 134 cases per 100,000 persons, resulting in 10.8 million illnesses and 1.25 million deaths [1]. Over the last 20 years, the incidence rate in Canada has remained mostly stable between 4.6 and 5.1 cases per 100,000 persons [3,4,5]. Locally in Newfoundland and Labrador, the prevalence remains below 5 cases per 100,000 persons [3,4,5]; however, outbreaks in the Inuit population of Nunatsiavut have reported incidence rates exceeding 200 cases per 100,000 persons [6].

While nontuberculous mycobacteria (NTM) infections do not carry the same public health implications as TB, differentiating between TB and NTM in AFB smear-positive specimens impacts the subsequent clinical management. NTM management is further complicated by the need to differentiate between true infections versus colonization, which is still a challenge in clinical decision making [7].

Early detection of TB is highlighted as one of the primary goals in TB elimination strategies, both globally and in Canada [8,9]. Current laboratory diagnostic approaches for clinical specimens include smear microscopy, nucleic acid amplification tests (NAATs), and culture. According to the Canadian Tuberculosis Standards, smear microscopy and culture are recommended for all samples, while NAATs are recommended for all smear-positive samples, and for smear-negative ones, upon request by physicians or public health [10]. In general, NAATs are reported with a sensitivity ranging from 60% in saliva specimens [11] to as high as 90% in respiratory specimens such as sputum, trach aspirate, and BAL [12,13].

Newfoundland and Labrador (NL) is a Canadian province with a population of 545,579 located on the northeastern seaboard of North America. All mycobacteria testing is conducted at the Provincial Public Health and Microbiology Laboratory (PHML) on symptomatic suspicion of mycobacteria infection and for select asymptomatic patients. Asymptomatic screening of TB, including collection of respiratory samples, occurs in immigration screening and close contact follow-up of TB cases in accordance with the Guideline for Preventing the Transmission of *Mycobacterium tuberculosis* across the Continuum of Care [14], which applies the national guidelines to NL local epidemiology [10]. Positive cultures are referred to the National Reference Center for Mycobacteriology (NRCM) for confirmation and systematic antimicrobial susceptibility testing. The TB outbreak in NL in 2015–2018 has highlighted gaps in early TB detection, especially the Inuit region of Nunatsiavut. This led to a reassessment of the NAAT testing algorithm used at that time, where NAAT was only performed on AFB smear-positive samples, and smear-negative samples when specifically requested. In November 2019, in response to ongoing elevated transmission within NL, PHML introduced a new Panel approach, where all samples with enough volume are tested with an upfront NAAT for MTBC and *Mycobacterium* spp. (*Myco* spp.), in addition to smear microscopy and culture. This observational study uses a retrospective pre–post design to evaluate the impact of this algorithm change on NL TB diagnosis and treatment.

## 2. Materials and Methods

Specimens submitted for Mycobacterial examination were tested using florescent acid-fast bacilli (AFB) smear, solid and liquid culture, and real-time polymerase chain reaction (qPCR). Prior to 18 November 2019, all specimens were processed using the Reflex algorithm in which AFB smear and culture were performed on all specimens, while NAAT testing was performed on all AFB smear-positive samples, as well as AFB smear-negative samples when requested. Starting on 18 November 2019, a new Panel approach was implemented, in which all specimens with sufficient volume would be tested by a laboratory-developed multiplex real-time polymerase chain reaction (m-qPCR) targeting both MTBC and *Myco* spp., along with culture and smear.

### 2.1. Culture and AFB Smear

All respiratory specimens were decontaminated using the N-acetyl-l-cysteine (NALC)-NaOH method [15]. Sterile body fluids were concentrated by centrifugation where volume allowed, while sterile tissues were homogenized prior to smear microscopy, culture, and NAAT testing. Specimens were then resuspended in approximately 2 to 3 mL of phosphate buffer; this concentrate was then used for all testing, as indicated by the testing approach. A 0.5 mL aliquot was inoculated into a BD BACTEC Mycobacteria Growth Indicator Tube (MGIT) and incubated using the MGIT 960 automated mycobacterial detection system. Respiratory specimens were inoculated on Lowenstein–Jensen (LJ) medium slants, incubated at 37 °C room air for 8 weeks. Non-respiratory specimens were also inoculated on LJ slants, with and without iron supplement, and incubated at 30 °C in a room air incubator. If a culture was flagged positively by the MGIT system or showed growth on LJ, an AFB smear was performed. If the AFB smear was positive, the culture was referred out of province to the NRCM at the Canadian National Microbiology Laboratory (NML) for further identification due to lack of a containment level 3 laboratory, in line with the national biosafety directive [16]. Antimicrobial susceptibility, performed at NRCM at NML, was performed by liquid BACTEC MGIT 960 system (Becton Dickinson, Sparks, MD, USA) testing [17].

### 2.2. Molecular Diagnostics

Molecular testing was performed as indicated per testing algorithm with two different assays. Prior to November 2019, the Reflex algorithm was employed using the Roche Amplicor MTB test, per manufacturer’s instructions (Roche Diagnostic Systems, Somerville, NJ, USA). Briefly, 0.1 mL of the pre-processed respiratory sample went through extraction per kit protocol. Non-respiratory samples were not processed. An amount of 50 μL of the extracted eluate mixed with 50 µL of master mix was used to set up qPCR reactions. The samples were then placed on the Roche Cobas TaqMan 48 for amplification and detection utilizing manufacturer instructions for settings and interpretations.

In the Panel approach implemented after November 2019, a laboratory-developed test (LDT) using the real-time multiplex MTBC/*Myco* spp. qPCR assay (m-qPCR) was designed and implemented. A volume of 0.6 mL of concentrated or homogenized samples was placed in a 2.0 mL microcentrifuge tube containing 100 µL of 0.1 mm silicon beads, 10 µL of proteinase K, and 10 µL of diluted T4 bacteriophage. The mixture was then incubated at 65 °C for 15 min to further liquify samples, followed by heat inactivation and lysis by submersion in boiling water for 25 min (long-term validated inactivation protocol). Samples then underwent mechanical lysis on a Digital Disruptor Genie (Scientific Industries, Inc., Bohemia, NY, USA) at 3000 RPM for 2 min. The subsequent lysate was then extracted using the MagnaPure Compact system (Roche Diagnostics GmbH, Mannheim, Germany) with 400 µL of samples to produce 50 µL of eluate.

Eluates were then used to perform m-qPCR on the LightCycler 480 II (Roche Diagnostics GmbH, Germany), using 20 µL of eluate with 30 µL of master mix in duplicate. Master mix was prepared with PrimeTime™ Gene Expression Master Mix, primer, and probes listed in Table 1 (Integrated DNA Technologies, Coralville, IA, USA). The m-qPCR assay was designed with the following DNA targets: IS6110 sequence for MTBC, an internal transcribed spacer (ITS) region specific to the Mycobacteria genus, T4 bacteriophage as an exogenous control, and a glyceraldehyde-3-phosphate dehydrogenase (GAPDH) as an endogenous control. The primers and probes were adapted from previously published works or, in the case of T4 and IS6110, developed at the National Microbiology Laboratory, Winnipeg, Canada. The 4-color real-time PCR assay was performed in a touchdown fashion, with a final annealing temperature of 64 °C performed over 50 total cycles; complete instrument settings can be found in Supplementary Table S1. A sample needed to have a positive amplification curve in duplicate to be considered positive. MTBC samples with a weakly positive cycle threshold (Ct) > 35.0 on only one sample were repeated to confirm positivity in the samples processed after March 2020. This change was implemented after 1 false-positive LDT m-qPCR result. In the Panel algorithm cohort, there were 2 other positive cases between 18 November 2019 and March 2020; both had Ct < 25.0 and would not have been impacted by this change. *Myco* spp. was considered positive only if positive in duplicate with a Ct ≤ 35.0 by the 2nd derivative maximum analysis method. *Myco* spp. specimens positive with Ct > 35.0–<39.0 were considered inconclusive. Further information on LDT m-qPCR method performance is located in Supplementary Table S2.

### 2.3. Data Collection and Analysis

Patient data were pulled retrospectively from laboratory information systems and provincial pharmacy databases. The provincial pharmacy database was implemented in 2017 and limited the retrospective data from that point onward. Both laboratory data and treatment data were pulled from 1 January 2017 to 31 December 2023. Patient’s data were excluded if there was an insufficient sample for all the tests indicated per the algorithm or if their treatment information was unable to be obtained through the databases (non-resident, death before treatment, or treated outside of province). The number for each anatomical source of positive cases is indicated in Supplementary Table S3. Patient demographics across the two periods were compared using the following statistical methods: the non-parametric Mann–Whitney U test was applied to assess age distribution for all groups; the Chi-squared test was used to compare total patients’ sex; and for MTBC/NTM patients’ sex distribution, Fisher’s Exact test was used due to the low sample number. The Mann–Whitney U test was calculated in BioRender, using R version 4.2.2, while the Chi-squared test and Fisher’s Exact test were calculated in Microsoft Excel (version 2508).

Presented in Table 2, this study analyzed results from 4578 patients, including 2507 in the Reflex algorithm period, with 33 cases of MTBC and 40 cases of NTM, and 2071 in the Panel approach period, including 30 cases of MTBC and 64 cases of NTM. Significant differences in demographics were observed in the age in both MTBC cases and total cases between the two cohorts.

To compare the diagnostic performance of the AFB smear, LDT m-qPCR, and culture assays, the following metrics were calculated: sensitivity, specificity, positive predictive value (PPV), and negative predictive value (NPV). For MTBC, clinical diagnosis determined through electronic chart review by licensed medical microbiologists served as a gold standard. Clinical diagnosis was determined by the most responsible physician for the patients’ care, guided by established criteria and national standards, which included assessment of clinical symptoms (e.g., cough, fever, weight loss, night sweats, etc.), epidemiological risk factors, such as close contact of a known case, microbiology findings, radiographic presentation, and clinical history, including previous TB infection and/or disease. For NTM, culture was used as the gold standard. *Myco* spp. target detection was not separately captured in the laboratory information system when MTBC was already positive, so it could not be utilized for accuracy calculations. *Myco* spp. inconclusive results were treated as positive results for the purpose of accuracy calculations. Assays were compared statistically by the McNemar test, performed in Microsoft Excel.

To evaluate the potential impact of the various assays on treatment decisions—and to determine whether a pre- vs. post-implementation comparison was appropriate—the timing of treatment during the post-implementation period was analyzed. Rapid diagnostics for the purpose of this study are defined as AFB smear and LDT m-qPCR. Often available within the same day or within 24 h of each other, it is not possible to determine retrospectively if a physician is changing care based on positive AFB smear or positive LDT m-qPCR; thus, they are grouped together for the following categorization. Cases were categorized as follows based on when treatment was initiated:

- Empiric: Treatment began before any diagnostic results were available.
- Rapid Diagnostics: Treatment was initiated after rapid diagnostics, smear, and/or LDT m-qPCR, results but before culture results were available.
- Culture-Based: Treatment was started after culture results were available.

To assess the impact of the Panel approach vs. Reflex algorithm on MTBC diagnosis, the time from specimen collection to MTBC specific diagnosis was calculated. This was categorized further by AFB smear status for each of the groups. Additionally, to account the fact that NL PHML refers all positive cultures, after growth of AFB is confirmed by microscopy, to the reference centre at the National Microbiology Laboratory (NML) in Winnipeg, MB, Canada, a modelling analysis was performed. The impact of the Panel approach vs. Reflex algorithm if there was rapid culture identification of MTBC was performed through molecular or antigen techniques, such as the Abbott Bioline TB Ag MPT64 test, on the day that mycobacterium were observed on the culture media [20].

To ensure changes to the timing of laboratory diagnosis correlated to changes to timing of treatment, the days from sample collection to treatment were obtained. The timing of treatment was considered to be the same day that pharmacy dispensed the medication for both Reflex algorithm and Panel approach. Statistical significance for time to diagnosis and time to treatment was assessed by Mann-Whitney U test to account for the non-normal distributed data and was performed within BioRender software for sample sets with *n* ≥ 30. For sample sets that have a smaller sample size Kruskal-Wallis statistical test was utilized.

The cost was calculated per specimen for the median batch size of four specimens and a negative process control. For the LDT m-qPCR method, only the cost of a qPCR-positive control was also included. Cost reflected here represents NL PHML prices in Canadian dollars (CAD). The timing of hands-on time of the assay was performed on three separate occasions, and the average per specimen time was obtained. Utilizing current labour prices, the cost of labour per specimen was obtained with the LDT m-qPCR method and the commercial comparator, the Cepheid GeneXpert MTB/RIF assay [21]. The current Canadian Tuberculosis Standards conditionally recommend testing one sample in smear-negative patients be performed upon request [10]. As a cost mitigation strategy, a retrospective analysis of the sensitivity of only testing one, two, or all specimens was calculated. This was calculated by utilizing the first, first and second, or all specimen results by collection time.

## 3. Results

### 3.1. Diagnostic Assay Performance

As shown in Table 3, the diagnostic accuracy of the LDT m-qPCR for MTBC determination was similar to that of the culture, with differences not reaching significance (*p* = 0.32), and superior to the AFB smear (*p* < 0.0001). The accuracy of the *Myco* spp. LDT m-qPCR for the NTM result was significantly lower than the culture (*p* < 0.001), but an improvement compared with the AFB smear (*p* = 0.002), when inconclusive results were excluded. Culture remained the most specific test for active disease detection for both MTBC and NTM. For MTBC LDT m-qPCR discordant results, one patient was falsely negative by m-qPCR, with a positive culture on day 42 only on LJ; it is important to mention that liquid culture on the BACTEC MGIT 960 was negative, and the AFB smear was negative. For false-positive results, one patient was weakly positive with a Ct value of 39.4, likely due to control contamination. After the introduction of the policy of repeating any weak positive if only one specimen was positive at this strength, no further false positives due to contamination were observed. The other two false-positive MTBC m-qPCR results were from patients who had completed TB treatment more than four years earlier, and were attributed to the presence of non-viable TB DNA. If culture is used as a gold standard instead of clinical diagnosis for MTBC, then the sensitivity, specificity, PPV, and NPV of LDT m-qPCR changes from 96.7%, 99.8%, 90.6%, and 99.95% to 96.2%, 99.7%, 81.3%, and 99.95%, thereby maintaining a non-significant difference from the culture (*p* = 0.125) and a significant difference from the smear (*p* < 0.0001).

### 3.2. Impact of Panel Approach on Case Management

Table 4 summarizes the impact of the Panel approach on treatment for MTBC and NTM. In 94% of MTBC cases, treatment was initiated based on rapid diagnostic results, including 57% of patients treated solely based on the positive MTBC LDT m-qPCR result. For NTM cases, 70% received no antibiotic treatment, and 28% were treated based on positive culture results. Only one patient (2%) had treatment initiated based on rapid diagnostics, of which both the smear and qPCR were positive. Therefore, due to the limited impact of the *Myco* spp. component of the m-qPCR assay, pre–post analysis of diagnosis and treatment was only conducted on MTBC cases, not including NTM cases.

### 3.3. Days to Diagnosis for MTBC

**Figure 1:**
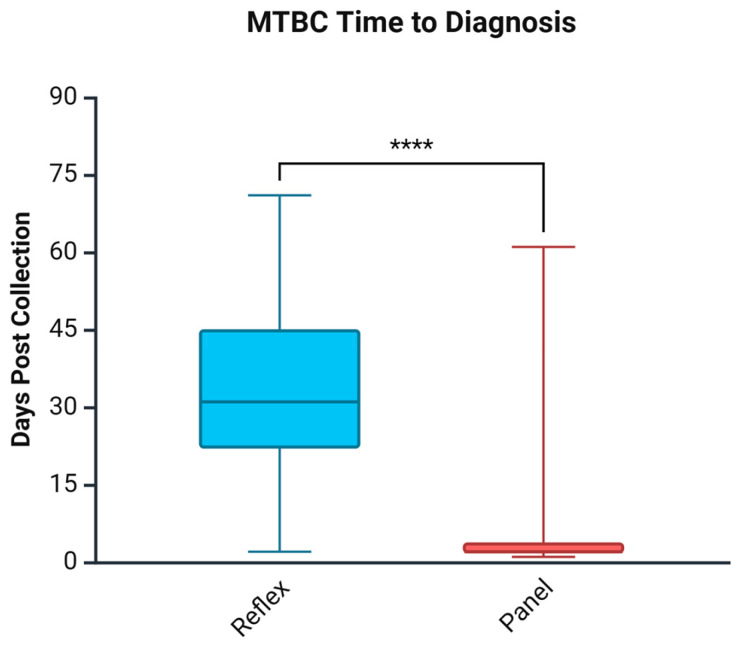
Days to MTBC diagnosis comparing the Reflex algorithm pre-cohort and the Panel approach post-cohort. **** *p* < 0.0001. Created in Biorender. Needle, R. (2025) https://BioRender.com (accessed on 3 March 2025).

As shown in Figure 1, compared to the Reflex cohort, the Panel approach significantly shortened the time to MTBC diagnosis by 29 days. Days to diagnosis in the Reflex algorithm was a median of 31 days; however, in the Panel approach, the time was reduced to 2 days. To address the confounding factor of referral identification, Table 5 shows that, even in laboratories with rapid culture identification of MTBC, the Panel approach still yields a statistically significant reduction in time to diagnosis by 14 days (*p* = 0.003). Further categorization of results by AFB smear status demonstrated that most of the improvement was seen in AFB smear-negative cases, with a reduction of 28 days (*p* < 0.001). AFB smear-positive groups were reduced by a non-significant amount of 4 days (*p* = 0.08); the result should be interpreted with caution given the low sample numbers.

### 3.4. Days to Treatment for MTBC

**Figure 2:**
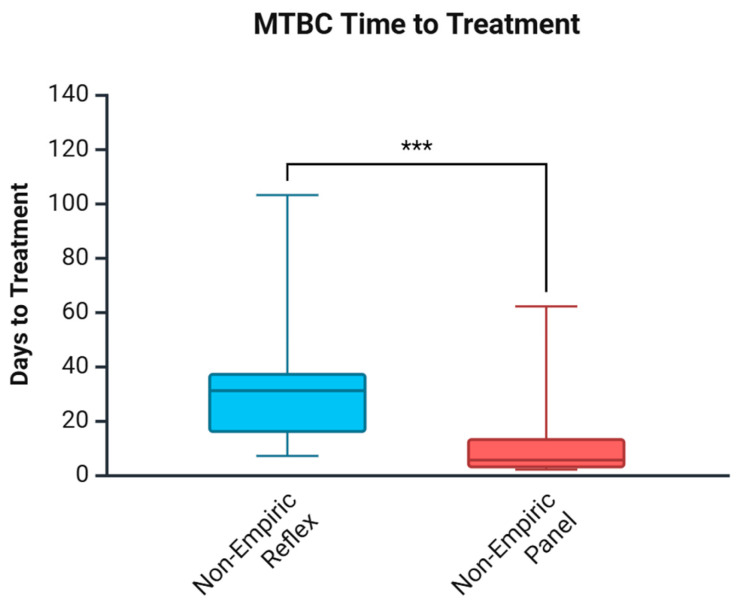
Days to MTBC treatment comparing the Reflex algorithm pre-cohort and the Panel approach post-cohort. *** *p* < 0.001. Created in Biorender. Needle, R. (2025) https://BioRender.com (accessed on 3 March 2025).

Table 5 and Figure 2 show the days to treatment for MTBC, comparing the impact of the Panel approach vs. the Reflex algorithm. In agreement with the findings for the time to diagnosis, the Panel approach reduced the time to treatment by 25.5 days among non-empirically treated patients. From collection to diagnosis was a median of 2 days, while from diagnosis to treatment was an added 3.5 days in the post-cohort in non-empirically treated patients. This was different in the pre-cohort, where the median collection to diagnosis was 31 days; however, diagnosis to treatment added 0 days in non-empirically treated patients. Categorization of time to treatment for non-empirically treated patients based on AFB smear results provided similar results to time to diagnosis. AFB smear-positive cases did not differ significantly between cohorts; however, AFB smear-negative cases treated non-empirically saw a reduction of 24.5 days (*p* = 0.004).

### 3.5. Impact of Panel Approach on Cost

Sustainability is an important aspect of any clinical diagnostic algorithm. The Panel approach, by design, increased molecular testing from 5–20 specimens per year to 1500–2000 specimens per year, and this testing volume increase comes at a cost. Annual labor cost did not increase with the Panel approach; however, the cost of labor, as defined by hands-on time, was calculated to be CAD 11.36 per specimen (Table 6). Comparatively some commercial molecular platforms, such as Cepheid GeneXpert MTB/RIF, offer less hands-on time but come with an increased material cost [22].

One way of mitigating the cost increase is to limit the testing to one specimen out of multiple collections per patient. As shown in Table 7, testing only the first specimen may be sufficient for maintaining high diagnostic sensitivity while optimizing resource utilization. Statistical analysis between all groups yielded a *p*-value of 1.0, showing no significant difference in sensitivity between testing the first specimen and all specimens in a diagnostic set. Testing the first sample based on the collection time in the diagnostic set of a new query TB case can be a method to reduce the material cost, particularly when more expensive reagents are utilized, while still maintaining a higher sensitivity than the 36.7% sensitivity of the AFB smear on MTBC cases. After the initial diagnosis, the MTBC m-qPCR assay and other DNA molecular assays will remain positive long even after the infection has resolved; therefore, repeat molecular testing provides little benefit (Figure S1). In the four-year post-implementation cohort, MTBC DNA remained detectable in a majority of samples for up to 6 months, and it decreased to less than 10% after 1 year.

## 4. Discussion

### 4.1. Diagnostic Assay Performance

The MTBC LDT m-qPCR demonstrated high sensitivity (96.7%) and specificity (99.8%), which is comparable to the performance of culture (90% sensitivity and 100% specificity). Meanwhile, the high PPV (90.6%) and NPV (99.95%) indicate the reliability of using the test to rule out disease. These findings support the use of molecular assays as the initial diagnostic test within the Panel approach in our setting.

For *Myco* spp. detection at the genus level, the assay showed a sensitivity (47.6%) higher than the smear for NTM (9.4%), but much lower than culture (100%). Although the high NPV (98.3%) supports the test as a tool to rule out disease, the low PPV (66.2%) limits its clinical utility as a rapid diagnostic method for NTM disease in a low-prevalence population.

### 4.2. Impact on Case Management

This study shows the most significant gain in the Panel approach in being able to detect TB cases at an early phase, which is critical for interrupting community transmission. In our non-empirically treated patients in the Panel approach (Table 4), 18 of 29 MTBC patients (62%) had a negative AFB smear and would have had no NAAT testing if a Reflex algorithm was used. This would have led to missed opportunities for early detection and treatment among this patient population. The same is not true for NTM disease, where rapid testing seemed to only impact the treatment of one patient (2%). NTM management guidelines are based upon radiologic evidence of disease, one or two positive cultures with the same medically-relevant species, depending on the source, with symptom presentation and progression to diagnose disease vs. colonization [23]. Our findings agree with the guidelines that rapid diagnostics, especially the addition of the genus-level LDT m-qPCR *Myco* spp. result, does not routinely impact the case management of NTM disease.

Our data demonstrated that implementing the Panel approach significantly improved TB diagnosis and treatment initiation compared to the Reflex algorithm. Among the MTBC patients, time to diagnosis was reduced by a median of 29 days in NL, with the greatest benefit seen among smear-negative cases (28 days). Similarly, time to treatment was shortened by a median of 25.5 days, with the greatest impact in smear-negative cases, at 24.5 days, as shown in Figure 2 and Table 5. This highlights the benefit of implementing universal rapid molecular testing, particularly in a setting like our province, with a low but heterogeneous TB burden, including high-risk Indigenous populations and foreign-born individuals. Finally, the referral process for culture confirmation to NML likely extended diagnostic timelines in NL. This potentially overestimates the impact of the Panel approach for health systems that have rapid culture identification locally. However, our modelling analysis confirmed that, even with rapid in-house culture identification, upfront NAAT still reduced the time to diagnosis by 14 days. Rapid in-house culture identification of MTBC is only one factor that laboratories should consider; other factors may also impact the time to diagnosis decrease, such as smear positivity rate.

In contrast, the impact on non-empirically treated, smear-positive patients was not statistically significant. The median time to diagnosis was 2 days in the Panel cohort compared to 6 days in the Reflex cohort (*p* = 0.08). Similarly, the time to treatment was reduced by just 3 days (4 days in the Panel cohort vs. 7 days in the Reflex cohort; *p* = 0.610). This lack of statistical significance is likely due to the small sample size and small improvement from testing NAAT in a reflex fashion, performed within the days following a positive smear vs. testing on the initial day. Other factors in a retrospective study could also have an impact, such as referral patterns and clinical decision making.

### 4.3. Cost and Operational Considerations

Our cost analysis (Table 6) showed that, while there are cost increases in the Panel approach, these can be mitigated. Restricting the test to the first collected sputum specimen reduces the cost while maintaining the high test diagnostic sensitivity, at 94.7%, for sputum sample MTBC detection. Laboratories must be cautious of this approach, given the inherent risk in missing positive cases, but most of the initial benefit of universal NAAT testing would remain. Compared to commercial assays, which usually have less labour cost but higher material costs, our LDT q-PCR demonstrated a reduced material cost, increased hands-on time, and unchanged annual staff cost in our setting of 1500–2000 tests annually with a low disease burden.

The AFB smear cannot be replaced entirely by NAAT tests for MTBC and NTM, especially during treatment monitoring. Consistent with other published data, this assay can only be used for diagnostics and does not show a high correlation in treatment monitoring (see Figure S2). The implication of different DNA Ct values is complicated to interpret due to mixtures of viable and non-viable organisms, specimen quality, specimen type, technical variation, and many other factors.

Feasibility of performing additional NAAT testing for MTBC, with the related cost increases, must be weighed by the priorities of the health care system. In NL, the investment was deemed prudent to expand outbreak management and outbreak prevention. Neither using the LDT to decrease material costs nor testing only the first sample of a patient’s samples will produce cost savings for the Panel approach in the laboratory; however, depending on the priorities, the change can be cost-effective. Davis et al., for instance, showed that rapid MTBC GeneXpert testing can reduce unnecessary empiric TB therapy, potentially reducing system costs [24]. Laboratories, especially in low-resource settings, must weigh these factors in the context of local epidemiology and resource availability.

### 4.4. Limitations and Confounders

This study has several limitations, including its retrospective observation design, which may introduce bias from unmeasured variables such as changes in clinician practices over time, specifically, the difference in epidemiology of the tail end of the local outbreak in 2017–2019 versus a lower incidence rate period involving the COVID-19 pandemic 2020–2023. Evidence of these differences can be seen in the number of empirically treated patients in the Reflex algorithm cohort of eight compared to only one in the Panel approach cohort. The age was significantly different between the two cohorts, likely because of different at-risk populations with higher local transmission preceding the Reflex algorithm period. The positive case counts were also limited; therefore, matching patients’ demographics, or other factors to control for confounders, was not possible. The strength of the conclusion drawn is also limited by the number of positive cases that could be assessed.

Using clinical diagnosis instead of culture as the gold standard in this study for MTBC disease may have biased test accuracy characteristics. Culture is highly specific; however, it could produce false-negative results if the mycobacterial load is low in paucibacillary infection or poor-quality specimens. In this case, true-positive TB cases detected by the molecular method may be deemed as false positives. While the performance characteristics of the LDT m-qPCR MTBC assay differ slightly when using culture as the gold standard, the conclusions that LDT m-qPCR MTBC is a significant improvement compared to AFB smear, and the difference between culture and LDT m-qPCR, did not reach statistically significance remain.

Additionally, the LDT m-qPCR assay does not detect resistance to rifampin, thus limiting its utility in certain situations. This limitation did not limit the impact in NL due to the antibiogram of MTBC (Table S4). However, outside of this study, the NL PHML has implemented the practice of sending all specimens positive for both AFB smear and LDT m-qPCR MTBC to a referral lab for rifampin genotypic susceptibility testing so to aid in the case management of potential multi-drug-resistant TB patients.

Finally, the low rate of NTM treatment initiation based on molecular results may reflect conservative clinical practices rather than a limitation of the assay itself, as NTM management often requires comprehensive clinical evaluation beyond microbiological detection. With the low rate of NTM cases treated (28%), with only one that was initiated after both AFB smear and m-qPCR *Myco* spp. were positive, a statistical comparison pre- vs. post- was not possible. Further study on the utility of molecular diagnostics in NTM disease is warranted given our significant limitations. NAAT for *Myco* spp. testing may still have value in targeted populations, especially if combined with species-specific discrimination or when restricted to extrapulmonary specimens.

### 4.5. Tuberculosis Control and Elimination

These findings support that the use of an initial NAAT in the Panel approach is essential to identify smear-negative cases, which leads to early clinical and public health responses. Although less contagious than smear-positive cases, smear-negative pulmonary TB patients still contribute to 10–20% of TB transmission [25,26]. A large-scale molecular epidemiology study in the Netherlands showed that smear-negative cases accounted for approximately 12.6% of secondary transmission [25]. In another eight-year cohort study conducted on 511 close contacts of pulmonary TB patients in Spain [26], 34.6% of contacts of smear-negative/GeneXpert-positive cases were found to have a positive TST/IGRA, compared to 46.2% among contacts of smear-positive cases.

The magnitude of the treatment delay we observed (≈4 weeks) is larger than that reported in the remote Xpert study by Alvarez et al. in the higher-incidence territory of Nunavut, Canada (median 10 days), but comparable to modelling from other low-incidence jurisdictions that incorporated the referral lag for culture confirmation [13,24]. These differences in impact are largely caused by the timing of sample collection in relation to disease progression and smear positivity. Studies and health systems with higher smear positivity rates will benefit less from this approach, whereas health care approaches that target at-risk patients that are asymptomatic or with mild, non-specific symptoms will likely benefit more.

Our findings endorse the World Health Organization (WHO) recommendation of phasing out smear microscopy and the utilization of NAAT tests as the initial test for all presumptive TB patients [7]. The current Canadian TB Standards continue to recommend NAAT on smear-positive specimens and selected smear-negative cases upon physicians’ request [10]. Our data suggest that this cautious stance may inadvertently prolong infectiousness and delay public health interventions, even in a low-incidence province where the per capita burden of TB is well below the national average.

Rapid microbiological confirmation of MTBC translated directly into treatment in 94% of infected patients during the post-implementation period, with over half started solely on the basis of a positive m-qPCR in an otherwise smear-negative sample. Earlier treatment initiation is known to reduce forward transmission, diminish the need for prolonged airborne isolation, and improve outcomes, particularly among contacts at a high risk of progression to severe disease. Importantly, for other jurisdictions with on-site rapid culture identification, the benefit persisted, indicating that upfront NAAT would still be advantageous for laboratories with full mycobacteriology capability.

Clinical management for NTM infection depends on clinical correlation, radiology, detection on repeated culture, and species-level identification/susceptibility results. Our results supported that genus-level detection of NTM was seldom actionable. For broad, low-prevalence populations, genus-level NAAT therefore appears to offer limited value on management decisions unless paired with downstream speciation or antimicrobial resistance testing.

## 5. Conclusions

Implementing NAAT testing for MTBC as an initial diagnostic tool led to a significant decrease in the time to diagnosis and time to treatment. The molecular assay can be performed on all diagnostic samples or the first sample collected in a set. Utilizing a molecular assay for genus-level identification of NTM had minimal impact on clinical management, suggesting its limited diagnostic utility in a broad population setting.

## Figures and Tables

**Table 1:** List of LDT m-qPCR primers and probes used.

Name	Sequence (5′ to 3′)	Final Conc.	Amplicon Length	Ref.
IS6110-F	CGGGACAACGCCGAATT	300	53	This Study
IS6110-R	GCCGACGCGGTCTTTAAAA	300		
IS6110-P	/56-FAM/CGAAGGGCGAACGC/3MGBEC/	200		
ITS-F	GGTGGGGTGTGGTGTTTGA	200	147	[18]
ITS-R	TGGATAGTGGTTGCGAGCA	200		
ITS-P	/5HEX/TGGATAGTGGTTGCGAGCA/3MGBEC/	250		
T4-F	ATCCAACACTAGGTTCTAACTGGACTG	250	164	This Study
T4-R	CGCTGTCATAGCAGCTTCAG	250		
T4-P	/56-ROXN/CGGAAATTTCTTCATCTTCCTCTGG/3IABRQSP/	250		
GAPDH-F	GGTGGTCTCCTCTGACTTCAACA	100	71	Modified from [19]
GAPDH-R	TGAGGGCAATGCCAGCC	100		
GAPDH-P	/5CY5/CCACTCCTC/TAO/CACCTTTGACGCTGG/3IABRQSP/	100		

**Table 2:** Summary of limited patient demographic information included in this study.

	Reflex Algorithm	Panel Approach	*p*-Value
Total Patients Male/Female Median Age in Years (Range)	2507	2071	
	1372/1135	1098/973	0.25
	68 (1–104)	67 (1–99)	<0.001
MTBC Patients Male/Female Median Age in Years (Range)	33	30	
	21/12	21/10	0.80
	32 (1–79)	42.5 (13–90)	0.04
NTM Patients	40	64	
Male/Female	10/30	18/46	0.82
Median Age in Years (Range)	67 (2–91)	67 (2–84)	0.86

**Table 3:** Accuracy of AFB smear, LDT m-qPCR, and culture during the post period of this study.

	Smear (MTBC, NTM)	LDT m-qPCR MTBC Result	LDT m-qPCR *Myco* spp. Result ^1^	Culture (MTBC, NTM)
True Positive	17 (11, 6)	29	17	91 (27, 64)
True Negative	1922	2038	1956	1978
False Positive	0	3	0	0
False Negative	77 (19, 58)	1	33	3 (3, 0)
True Inconclusive ^2^	N/A	N/A	13	N/A
False Inconclusive ^2^	N/A	N/A	19	N/A
Sensitivity	18.1% (36.7, 9.4)	96.7%	47.6%	96.8% (90.0, 100)
Specificity	100%	99.8%	99.0%	100%
PPV	100%	90.6%	61.2%	100%
NPV	96.1% (99.0, 97.1)	99.95%	98.3%	99.8% (99.9, 100)

^1^ Accuracy calculations exclude all MTBC-positive patients, as the *Myco* spp. target was not interpreted when IS6110 (the MTBC sequence target) was positive. ^2^ *Myco* spp. inconclusive results were considered to be positive for calculations of LDT m-qPCR performance characteristics. Legend: PPV: positive predictive value; NPV: negative predictive value. N/A: not applicable.

**Table 4:** Diagnostic assays impacting treatment initiation.

	Number of MTBC Cases (%)	Number of NTM Cases (%)
No Treatment	0 (0%)	45 (70%)
Empiric	1 (3%)	0 (0%)
m-qPCR+/Smear−	17 (57%)	0 (0%)
m-qPCR+/Smear+	11 (37%)	1 (2%)
m-qPCR−/Smear+	0 (0%)	0 (0%)
Culture	1 (3%)	18 (28%)

**Table 5:** Impact of time to diagnosis and time to treatment in the pre- and post-cohorts.

	Reflex Median (*n*, Range)	Panel Median (*n*, Range)	Difference	*p*-Value
Days to Diagnosis MTBC	31 (33, 2–71)	2 (30, 2–61)	29	*p* < 0.0001
Days to Diagnosis AFB+	6 (8, 2–23)	2 (11, 1–5)	4	*p* = 0.08
Days to Diagnosis AFB−	31 (25, 2–71)	3 (19, 1–61)	28	*p* < 0.0001
Days to Diagnosis w/Rapid Culture ID	16 (33, 1–59)	2 (30, 1–42)	14	*p* = 0.003
Day to Treatment, All Cases	18 (33, −1–103)	5 (30, 0–62)	13	*p* = 0.002
Days to Treatment, Non-Empiric Only	31 (25, 7–103)	5.5 (29, 2–62)	25.5	*p* < 0.001
Days to Treatment, AFB+, Non-Empiric Only	7 (5, 2–36)	4 (11, 2–20)	3	*p* = 0.610
Days to Treatment, AFB−, Non-Empiric Only	32 (20, 6–103)	7.5 (18, 2–62)	24.5	*p* = 0.004

**Table 6:** Cost profile of LDT m-qPCR compared to commercial assay.

Cost Components	LDT Assay (CAD)	Commercial Assay (CAD)
Materials	CAD 13.26	CAD 68.25
Labor	CAD 11.36	CAD 1.36
Total	CAD 23.62	CAD 69.61

**Table 7:** MTBC LDT m-qPCR sensitivity on MTBC detection using first, second, or all samples in multiple collection set.

	Sputum Sensitivity (95% CI)	Respiratory ^1^ Sensitivity (95% CI)
First Specimen Only	94.74 (73.97–99.87)	92.6% (75.71–99.09)
First and Second	94.74 (73.97–99.87)	96.3% (81.03–99.91)
All Specimens ^2^	94.74 (73.97–99.87)	96.3% (81.03–99.91)

^1^ Included in the specimen category: sputum, bronchial washings, bronchial lavages, and any other respiratory secretion specimens. ^2^ All specimens include all of the specimens received on the patients; 1 or more.

## Data Availability

The complete datasets presented in this article are not readily available because of privacy reasons. Requests to access a limited and anonymous dataset should be directed to Dr. Lei Jiao.

## Supplementary Materials

The following supporting information can be downloaded at: https://www.mdpi.com/article/10.3390/biomedicines13102416/s1, Table S1. LDT m-qPCR program; Figure S1. Positivity rate of various assays at different time points, with the initial point being date of initial diagnosis; Figure S2. LDT-qPCR cycle threshold value correlated with days to positivity of culture; Table S2. LDT m-qPCR validation characteristics overview; Table S3. MTBC and NTM case numbers by specimen source; Table S4. Antibiogram for MTBC 2017–2023 NL.

## Abbreviations

The following abbreviations are used in this manuscript:

NAAT	Nucleic acid amplification testing
MTBC	*Mycobacterium tuberculosis* complex
TB	Tuberculosis
NTM	Nontuberculous mycobacteria
NL	Newfoundland and Labrador
PHML	Provincial Public Health and Microbiology Laboratory
NRCM	National Reference Center for Mycobacteriology
*Myco* spp.	*Mycobacterium* spp.
AFB	Acid-fast bacilli
m-qPCR	Multiplex real-time polymerase chain reaction
NALC	N-acetyl-l-cysteine
MGIT	Mycobacteria Growth Indicator Tube
NML	National Microbiology Laboratory
LJ	Lowenstein–Jensen
LDT	Laboratory-developed test
ITS	Internal transcribed spacer
GAPDH	Glyceraldehyde-3-phosphate dehydrogenase
Ct	Cycle threshold
PPV	Positive predictive value
NPV	Negative predictive value
N/A	Not applicable
ID	Identification
