# Supplementary material for: Evaluating the Impact of a Molecular Diagnostic Algorithm on Tuberculosis and Nontuberculous Mycobacterial Infections in Newfoundland and Labrador, Canada
Source: Biomedicines. 2025 Oct 2;13(10):2416. doi: 10.3390/biomedicines13102416 (PMC12561976; doi:10.3390/biomedicines13102416)
Supplement: Supplementary file 1 [file biomedicines-13-02416-s001.zip › biomedicines-3815345-supplementary.pdf]

Supplementary Materials

Table S1. LDT m-qPCR program

Target/Secondary Target(˚C)	Hold (mm:ss)	Acquisition
**Hot Start -1 cycle**		
95	3:00	none
**Touchdown PCR-5 cycles, delay 3 cycles, decrease by 1˚C per cycle**		
95	00:10	none
66/64	01:00	none
**Amplification-45 cycles**		
95	00:10	none
64	01:00	Single[1]
**Cooling-1 cycle**		
40	00:30	none

[1]Detection setting for channels 465-510, 533-580, 533-610, 618-660 are melt factor=1, Quant Factor =10, Max integration Time=2 seconds

Table S2. LDT m-qPCR Validation Characteristics Overview

Characteristic	Experiment summary	LDT m-qPCR *Myco spp.*	LDT m-qPCR MTBC
qPCR Efficiency	1:10 dilution serires DNA 3x10^7 cps/rxn - 300 cps/rxn with 4 replicates	99.8%	98.2%
Limit of Detection	Dilution of known concentration of DNA at 3, 6, 12, 18.75, 37.5 cps/rxn with 20 replicates each, 95% Probit analysis	31.8 cps/rxn<br>(95% CI 22.1-45.1)<br>~200 CFU/mL	3.5 cps/rxn<br>(95% CI 2.7-4.6)<br>~3.5-28 CFU/mL[1]
Analytical Specificity	Following strains:<br>*M. tuberculosis* ATCC 27294<br>*M. kansasii* ATCC 12478<br>*M. fortuitum* ATCC 6841<br>*M. phlei* ATCC 11758<br>*M. smegmatis* ATCC 14468<br>*M. chelonae* ATCC 35752<br>*S. aureus* ATCC 25923<br>*K. pneumoniae* ATCC 33495<br>*S. pneumoniae* ATCC 6305<br>*Ps. aeruginosa* ATCC 27853<br>*Actinomyces spp.* clinical strain<br>*Nocardia spp.* clinical strain<br>*S. pneumoniae* clinical strain	Positive for all *Myco spp.*, negative for all others	Positive for only *M. tuberculosis*
Sensitivity	15 NTM, 26 MTBC, 84 negative retrospective (previously frozen) samples	60%	100%
Specificity		100%	100%

Percent Inhibition	Inhibition was analyzed on 125 clinical specimen using exogenous and endogenous controls	0%	0%
Within Run Precision	300 cps/rxn positive control repeated in 5 runs with 5 replicates each run with precision values calculated in EP evaluator software	0.3% CV	0.3% CV
Between Run Precision		0.7% CV	0.5%CV
Reagent Stability	Mastermix was aliquoted into 5 tubes, tubes underwent 0, 1, 2, 3, 4, 5, 6,or 7 freeze thaw cycles. Mastermix was then run in duplicate to determine ΔCt	1)ΔCt=0.2<br>2)ΔCt=-0.1<br>3)ΔCt=0.5<br>4) ΔCt=0.4<br>5) ΔCt=0.5<br>6) ΔCt=0.4<br>7) ΔCt=0.5	1)ΔCt=0.1<br>2)ΔCt=0.3<br>3)ΔCt=0.4<br>4) ΔCt=0.5<br>5) ΔCt=0.5<br>6) ΔCt=0.4<br>7)ΔCt=0.5
Sample Stability	Positive and Negative pools of patient samples were stored at 3 different conditions:<br>1) 7 days at 2-8°C<br>2) 14 days at 2-8°C<br>3) 3 freeze thaw cycles at -20°C<br><br>Compared with same sample pools sampled ran on day 0, all samples ran in replicates of 4	1)ΔCt=0.4<br>2)ΔCt=0.4<br>3)ΔCt=-0.16	1)ΔCt=0.2<br>2)ΔCt=0.3<br>3)ΔCt=-0.3

Legend: cps/rxn= copies/reaction, ΔCt= Change in average Ct values, CV= Coefficient of variation

[1] IS6110 MTBC target is a multi-copy gene thus limit of detection will change based on number of copies in the strain. Approximate CFU/mL strain is based on local strain with ~8 copies per bacterium. Some southeast Asian strains have been reported to not contain IS6110 and caution should be taken on false negative results because of this.

Table S3. MTBC and NTM Cases Numbers by Specimen Source

Source	Reflex Algorithm		Panel Approach	
	MTBC	NTM	MTBC	NTM
Sputum	22	10	18	14
Bronchial Wash	5	16	6	24
Bronchoalveolar Lavage	1	12	1	19
Tissue	2	1	4	5
Sterile Body Fluid	1	0	1	0
Tracheal Aspirate	0	0	0	1
Gastric Fluid	1	0	0	0
Urine	1	0	0	0
Stool	0	1	0	0
**Total**	33	40	30	64

Figure S1. Positivity rate of various assays at different time points with the initial point being date of initial diagnosis

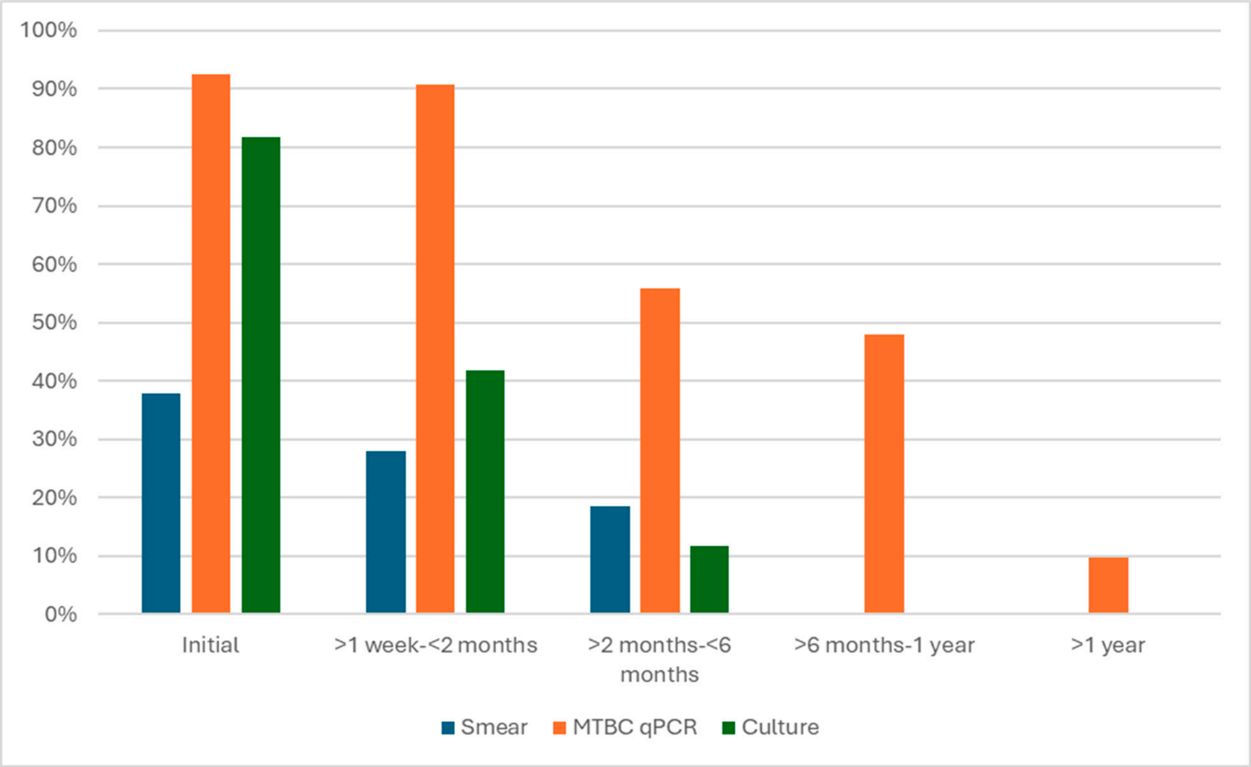

Figure S2. LDT-qPCR Cycle Threshold value correlated with Days to positivity of Culture.

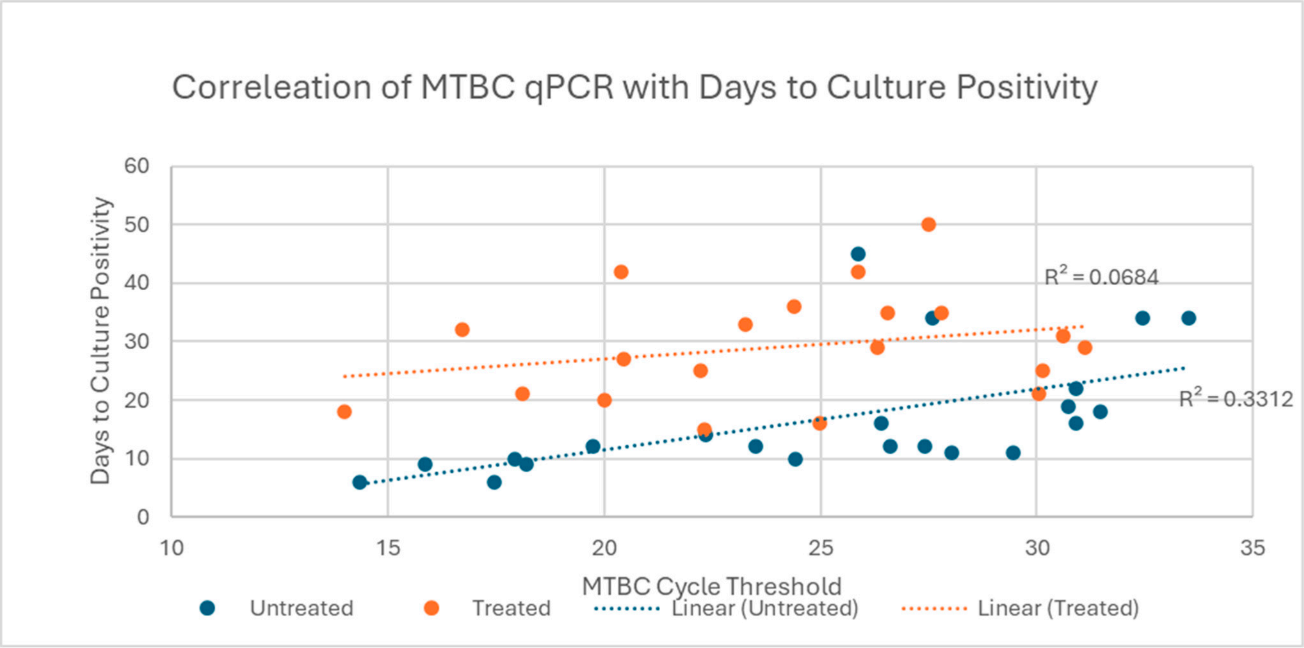

Untreated patient MTBC specimens $R^2 = 0.3312$, Patient undergoing treatment $R^2 = 0.0684$.

Table S4. Antibiogram for MTBC 2017-2023 NL

Antibiotic	Isoniazid	Rifampin	Ethambutol	Pyrazinamide
Sensitive	61	63	63	62
Intermediate	0	0	0	0
Resistant	2	0	0	1
Percent Susceptible	96.8%	100%	100%	98.4%
